# Challenges in diagnosing pseudomyxoma peritonei in a hepatitis B patient: A case report

**DOI:** 10.1097/MD.0000000000042298

**Published:** 2025-04-25

**Authors:** Yan Xing, Xuguang Jiang, Xiang Li, Xuan Li, Haifeng Liu

**Affiliations:** a Department of Ultrasound, Zaozhuang Municipal Hospital, Zaozhuang, China; b Department of Imaging, Zaozhuang Municipal Hospital, Zaozhuang, China.

**Keywords:** chronic hepatitis B, cirrhosis, computed tomography, magnetic resonance imaging, pseudomyxoma peritonei

## Abstract

**Rationale:**

Pseudomyxoma peritonei (PMP) is a rare condition, typically associated with the rupture of an appendiceal mucinous tumor. Due to its rarity and the complexity of its pathophysiology, PMP presents a significant diagnostic challenge.

**Patient concerns:**

A 59-year-old woman presented with a 6-month history of abdominal distension, anorexia, and significant weight loss. Her medical history was notable for chronic hepatitis B. She was initially treated for suspected cirrhosis, but her symptoms did not improve and therefore further diagnostic evaluation was needed.

**Diagnosis:**

Initial imaging, including liver magnetic resonance imaging (MRI) and abdominal ultrasound, showed irregular liver margins and ascites, consistent with cirrhosis. However, subsequent diagnostic tests, including uterine MRI, positron emission tomography–computed tomography, and abdominal ultrasound, revealed features suggestive of PMP. Immunohistochemistry and histopathological examination of tissue samples confirmed low-grade mucinous adenocarcinoma of gastrointestinal origin, specifically from the appendix.

**Interventions:**

The patient underwent cytoreductive surgery, and postoperative pathology confirmed mucinous adenocarcinoma originating from the appendix. The patient was also treated with intraperitoneal hyperthermic chemotherapy.

**Outcome:**

The final diagnosis was PMP.

**Lessons:**

This case illustrates a rare presentation of PMP in a patient with co-existing hepatitis B, in which the initial diagnosis was biased towards cirrhosis: a more common cause of ascites in hepatitis B patients. This case highlights the importance of considering PMP in the differential diagnosis for patients presenting with unexplained ascites and abdominal distension.

## 1. Introduction and background

The term pseudomyxoma peritonei (PMP) was coined by Werth in 1884 to describe a rare disorder associated with ruptured mucinous tumors, primarily originating from the appendix.^[1]^ The complex pathophysiology of PMP remains incompletely understood. PMP cases are predominantly associated with ruptured AMN, encompassing both low-grade and high-grade mucinous tumors, and 40% of cases manifest as an unexplained increase in abdominal circumference.^[1,2]^ The incidence of PMP is estimated at approximately 1 to 2 cases per million, with a higher prevalence in women.^[3,4]^ Characterised by the gradual and continuous accumulation of gelatinous fluid in the peritoneal cavity, PMP may exhibit sparse and inconspicuous tumor cells.^[5,6]^ Typically, PMP becomes evident 5 to 30 years postprimary lesion, contributing to delayed diagnosis.^[1]^ Furthermore, PMP syndrome demonstrates diverse biological behavior and malignant potential, often leading to extensive mucinous ascites enveloping the abdomen and pelvis.^[7]^ If left untreated, patients may succumb to complications arising from accumulated mucinous ascites, inducing bowel obstruction, perforation and related sequelae caused by the tumor.^[8]^ Therefore, confirmatory diagnosis of PMP through computed tomography (CT), magnetic resonance imaging (MRI) and expert pathological examination is imperative before initiating treatment.^[9]^

This case report presents a patient with PMP primarily manifesting as ascites, complicated by a concurrent hepatitis B infection. The diagnostic process was challenging, largely due to the rarity of PMP and the inherent biases in clinical judgment influenced by the patient’s hepatitis B history. We believe this case report is of significant value, as the early course of “misdiagnosis” may offer critical insights for other clinicians.

## 2. Case presentation

The patient, a 59-year-old female, presented 6 months ago with abdominal distension, loss of appetite, and weight loss (approximately 5 kg). She denied experiencing abdominal pain, diarrhea, nausea, vomiting, fever, headache, cough, sputum production, chest tightness, or chest pain. Three months ago, her symptoms of abdominal distension worsened. She was diagnosed with hepatitis B surface antigen positivity 20 years ago and has since received antiviral therapy. An upper abdominal CT scan revealed irregular liver margins suggestive of cirrhosis, accompanied by ascites. Subsequently, she presented to the Hepatology Department at our hospital for treatment. She had a history of gastrointestinal bleeding without systematic treatment. On physical examination, the abdominal wall was soft, without tenderness or abdominal wall varices, but positive shifting dullness. Following admission, the patient was administered liver-protective, diuretic and antiviral treatments, resulting in slight symptom relief but persistent anorexia and physical decline. Infectious disease screening revealed that hepatitis B surface antigen, hepatitis B e antigen, and hepatitis B core antibody were all positive. Tumour markers indicated elevated CEA (8.7 ng/mL), CA125 (115.1 U/mL) and CA199 (226.9 U/mL).

**Figure 1.**
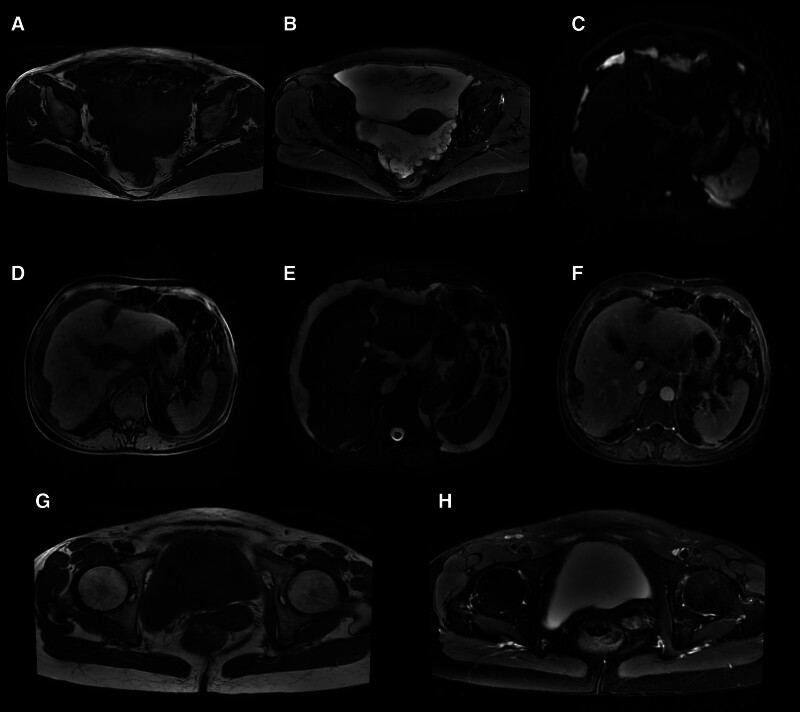
Representative imaging findings. (A) Ascites shows low signal intensity on T1WI. (B) Ascites shows high signal intensity on T2WI. (C) Irregular liver margins and perihepatic abnormal signals with high signal intensity on DWI. (D) Irregular liver margins and perihepatic abnormal signals with low signal intensity on T1WI. (E) Irregular liver margins and abnormal perihepatic signals with high signal intensity on T2WI. (F) Perihepatic abnormal signals with delayed-phase enhancement. (G) Abnormal cervical signal with slightly high intensity on T1WI. (H) Abnormal cervical signal with high intensity on T2WI. DWI = diffusion-weighted imaging, T1WI = T1-weighted imaging, T2WI = T2-weighted imaging.

**Figure 2.**
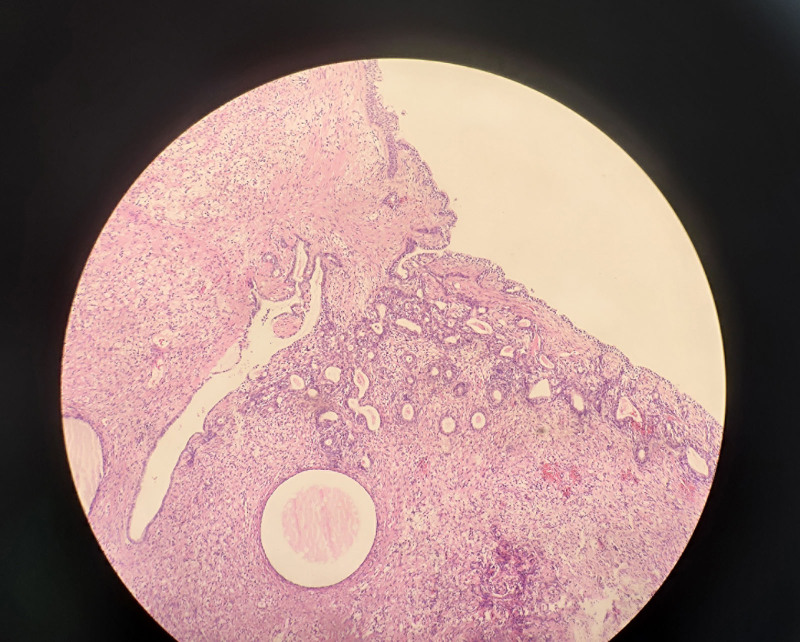
Cervical polyp, inflammatory hyperplasia of cervical interstitium and glands.

**Figure 3.**
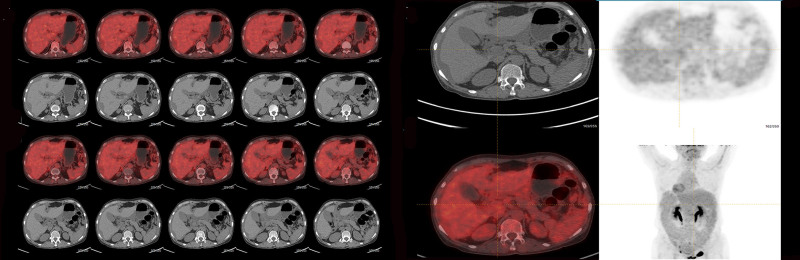
PET/CT scan showed irregular liver shape, widened portal vein, about 1.5 cm in diameter, and liquid density shadow was seen around the liver. PET/CT = positron emission tomography–computed tomography.

**Figure 4.**
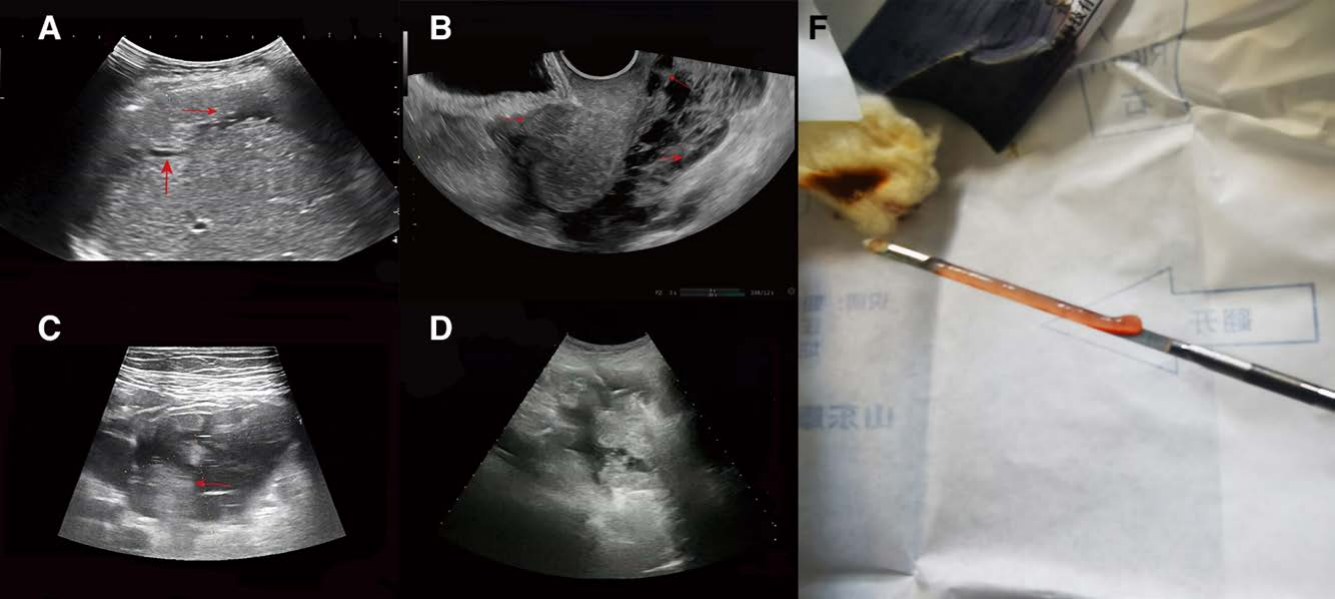
(A) Thickening of the perihepatic peritoneum, with finger-pressure signs visible on the surface of the liver. (B)Nodules on the plasma surface of the uterus, foveal fluid dark areas in the cysto-uterine fossa and the rectouterine pouch, and diffuse moderate echogenicity. (C) Hypoechoic mass in the ileocecal region with visible nodules. (D) Ultrasound-guided puncture. (E) Tissue strip.

**Figure 5.**
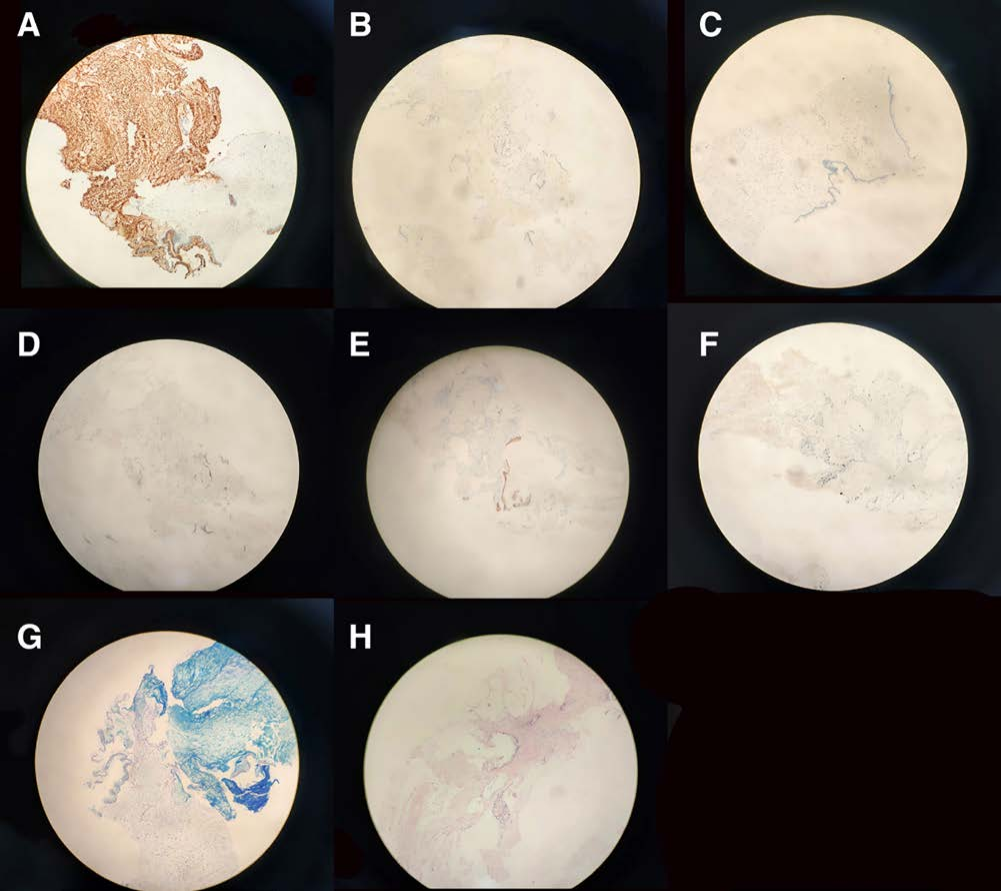
(A) MUC5AC (+). (B) MUC6 (−). (C) PAX-8 (−). (D) SATB2 (+). (E) Villin (+). (F) Ki-67 30% (+). (G) Special staining revealed AB-PAS-positive intracellular and extracellular mucin. (H) HE staining, large amount of mucus in the abdominal cavity with scattered tumor cells, most of which showed adherent growth with little cellular heterogeneity. AB-PAS = Alcian blue-periodic acid-Schiff, HE = hematoxylin and eosin, MUC5AC = mucin 5AC, MUC6 = mucin 6, PAX-8 = paired box gene 8, SATB2 = special AT-rich sequence-binding protein 2.

Liver MRI plain and dynamic enhancement scans revealed cirrhosis, ascites, gastroesophageal varices and a right lobe hepatic cyst (Fig. 1A–F). Uterine MRI identified abnormal cervical signals, accompanied by ascites, pelvic effusion, omental thickening and peritoneal thickening (Fig. 1G and H). Pathological findings from the excision of the cervical lesion indicated a polyp (Fig. 2). Positron emission tomography–computed tomography (PET/CT) demonstrated cirrhosis, portal hypertension and ascites, with no abnormal (18) F-2-fluoro-2-deoxy-d-glucose (FDG) metabolism in the uterus but a thickening of the omentum (Fig. 3). The tuberculosis bacillus antibody was negative, and the colonoscopy revealed no abnormalities. Ultrasound revealed thickening around the liver, spleen, and peritoneum, finger-like changes on the liver surface, and honeycomb-like ascites with diffuse moderate echogenicity. Additionally, a low-echoic mass (4.7 × 3.5 cm) with a wall thickness of approximately 0.2 cm and multiple medium-echo nodules (the largest being 1.7 × 1.4 cm) were identified in the ileocecal area, closely associated with the cecum, with inconspicuous blood flow signals (Fig. 4). Tissue samples were ultimately obtained through ultrasound-guided abdominal paracentesis. Immunohistochemistry results demonstrated positivity for Villin, special AT-rich sequence-binding protein 2 and mucin 5AC, and negativity for mucin 6 and paired box gene 8, with 30% positivity for Ki-67 (Fig. 5A–F). Special staining revealed Alcian blue-periodic acid-Schiff-positive intracellular and extracellular mucin 67 (Fig. 5G). The pathological report indicated a low-grade mucinous carcinoma, with immunohistochemistry confirming its gastrointestinal origin (Fig. 5H). The patient underwent cytoreductive surgery (CRS) at a higher-level hospital, and postoperative pathology revealed an appendiceal mucinous adenocarcinoma. Subsequently, the patient received hyperthermic intraperitoneal chemotherapy (HIPEC).

## 3. Discussion

Appendiceal mucinous neoplasms (AMN) represent <1% of all cancers and only 0.2% to 0.3% of appendectomy specimens.^[10]^ PMP, an even rarer condition, is characterized by the peritoneal implantation of tumor cells, leading to extensive intraperitoneal accumulation and redistribution of mucinous ascites.^[11]^ The primary etiology of PMP is AMN, with other rare causes including pancreatic, urachal and ovarian teratomas.^[12]^

In this case, both abdominal CT and MRI revealed an irregular liver edge and ascites. Given the patient’s history of hepatitis B and symptoms of abdominal distension, radiologists could easily prioritize cirrhotic ascites as the primary diagnosis. This assumption might mislead clinicians to pursue cirrhosis treatment as the initial approach, as cirrhosis is a common cause of ascites. To explore other sources of ascites, a dynamic MRI of the uterus revealed abnormal cervical signals, leading to cervical polyp confirmation through subsequent surgery. Meanwhile, dynamic MRI of the uterus suggested thickening of the greater omentum and peritoneum, raising the possibility of metastasis. Consequently, the PET/CT scan continued to identify “cirrhosis” as the primary diagnosis. This diagnostic bias in the imaging reports may result from several factors: the influence of the initial diagnosis on subsequent clinical decisions, potentially leading to anchoring bias. Furthermore, as PMP is a slow-growing tumor characterized by low cellular metabolic activity and a high mucin content that poorly absorbs FDG, it is susceptible to being overlooked in imaging studies. Although clinical diagnosis and radiological examinations are independent processes, there exists a possibility of an interaction between the primary physician’s initial diagnosis and the radiologist’s report, particularly among inexperienced radiologists. Moreover, the slow growth, low metabolic activity and high mucin content of PMP may contribute to its oversight in FDG-PET scans. Ultimately, a low-grade mucinous carcinoma from the appendix was diagnosed using abdominal ultrasonography-guided paracentesis, immunohistochemistry and pathology. Despite its low-grade cellular morphology and lack of invasiveness, this carcinoma exhibited malignant biological behavior with occasional lymph nodes or distant metastases, posing a diagnostic challenge.

Ultrasonography revealed significant ascites, peritoneal thickening, a honeycomb-like structure in certain areas and diffuse moderate echoes around the liver, spleen and uterine rectal and vesicouterine pouches. Additionally, the fluid wave sign on the liver surface further supported the diagnosis of PMP, characterized by the accumulation and redistribution of mucin within the peritoneal cavity.^[13]^ The mucin and tumor cells, moving with the flow of ascitic fluid, redistribute under the influence of lymphatic spaces and lymphatic cells, leading to the preferential accumulation of tumor cells in areas such as the pelvis, mesocolon, greater omentum and hepatic capsule. During mucin absorption, the epithelial cells are filtered out, resulting in the deposition of excessive gelatinous material.^[14]^

Ascites induced by PMP can be differentiated through ultrasound characteristics, distinguishing them from cirrhotic ascites, tuberculous ascites and carcinomatous ascites. Typically, cirrhotic ascites present as exudative, demonstrating good sonographic transmission and visible intestinal floating signs, along with features such as liver cirrhosis and gallbladder wall thickening.^[15]^ Tuberculous ascites can be accompanied by peritoneal thickening, with multiple fine reticular structures in the ascitic fluid, along with symptoms such as low fever and fatigue.^[16]^ Furthermore, carcinomatous ascites exhibit features like omental cakes and nodules; however, they lack mucinous characteristics, with the identification of the primary tumor aiding in differential diagnosis.^[15]^ Additionally, peritoneal mesothelioma generally manifests as either diffuse or nodular thickening of the omentum and peritoneum, often with ascites exhibiting or lacking septations.^[17]^

PMP primarily originates from AMN. Therefore, in cases presenting with mucinous ascites, particular attention should be paid to the appendiceal region to locate the primary lesion. Appendiceal mucinous tumors typically appear as single-chambered cystic masses in the right lower abdomen, exhibiting distinct ultrasound features like elongated or circular shape, onion-skin-like echogenicity and close association with the cecum. Notably, nodular structures within the appendiceal lumen can be indicative of malignant potential,^[18]^ with these nodules representing coagulated mucin and granulation tissue formed due to cancer cell proliferation.^[19]^ In summary, although appendiceal mucinous tumors concomitant with PMP are rare, their mucinous ascites present distinct ultrasound features that assist in identifying the appendiceal mucinous tumor as the primary lesion, thereby providing crucial ultrasonographic evidence for the diagnosis of PMP.

Currently, the standard treatment for PMP involves a combination of CRS and HIPEC. CRS aims to remove visible tumor deposits from the peritoneal surfaces, striving for complete cytoreduction, which is a crucial determinant of long-term survival outcomes. Following CRS, HIPEC is performed, which circulates heated chemotherapeutic agents within the peritoneal cavity to eliminate microscopic residual tumor cells, enhancing drug efficacy and reducing recurrence rate.^[20,21]^ Despite the effectiveness of CRS and HIPEC, systemic chemotherapy remains less effective due to the mucinous nature of PMP, which hinders drug penetration. However, systemic chemotherapy may still be utilized in cases where surgery is not feasible or for managing recurrent disease.^[22]^ Recent advancements are exploring the potential of targeted therapies, immunotherapies, and novel chemotherapeutic agents to improve treatment outcomes.^[22]^ Mucolytic agents, which can break down mucin, are being investigated to facilitate better drug delivery.^[23]^ Additionally, personalized treatment approaches based on molecular and genetic profiling of tumors hold promise for more effective management of PMP.^[23]^ In cases where surgery is not feasible, alleviating the patient’s discomfort and enhancing their quality of life becomes a crucial therapeutic approach. At recent follow-up, the patient was receiving intermittent symptomatic treatment with Chinese medicine and was able to independently manage her daily affairs.

## 4. Conclusion

This case highlights the importance of maintaining diagnostic vigilance when evaluating ascites, particularly in patients with common comorbidities such as hepatitis B. Ultrasound and ultrasound-guided aspiration proved essential in differentiating PMP from cirrhosis-related ascites, underscoring the value of direct evidence over clinical assumptions. Clinicians should remain alert to cognitive biases and adhere to standardized diagnostic protocols, especially when rare conditions are involved.

## Author contributions

**Conceptualization:** Yan Xing, Xuguang Jiang, Haifeng Liu.

**Data curation:** Haifeng Liu.

**Supervision:** Haifeng Liu.

**Validation:** Haifeng Liu.

**Visualization:** Yan Xing.

**Writing – original draft:** Yan Xing, Xuguang Jiang, Xuan Li, Haifeng Liu.

**Writing – review & editing:** Yan Xing, Xuguang Jiang, Xiang Li, Haifeng Liu.
